# Early detection and monitoring of hearing loss in vestibular migraine: Extended high-frequency hearing

**DOI:** 10.3389/fnagi.2022.1090322

**Published:** 2023-01-10

**Authors:** Zhaoqi Guo, Jun Wang, Dan Liu, E. Tian, Jingyu Chen, Weijia Kong, Sulin Zhang

**Affiliations:** ^1^Department of Otorhinolaryngology, Union Hospital, Tongji Medical College, Huazhong University of Science and Technology, Wuhan, Hubei, China; ^2^Union Hospital, Institute of Otorhinolaryngology, Tongji Medical College, Huazhong University of Science and Technology, Wuhan, China

**Keywords:** vertigo, vestibular migraine, hearing loss, extended high frequency, pure tone audiometry, prediction

## Abstract

**Background:**

Vestibular migraine (VM) presents mainly with recurrent vestibular symptoms and migraine. A great number of patients with VM have cochlea symptoms such as tinnitus, hearing loss.

**Methods:**

A cross-sectional study was conducted on patients with definite VM (dVM) and probable VM (pVM) who met the diagnostic criteria. Auditory-vestibular tests and psychological assessments were performed. Logistic regression was used to evaluate the predictive effect of EHF pure tone audiometry (PTA) for standard frequency (SF) hearing loss.

**Results:**

Fifteen patients with pVM and 22 patients with dVM were recruited. Overall, the two most vertigo types were vestibulo-visual symptoms (83.78%) and internal vertigo (54.05%). A vertigo attack persisted for <5 min in approximately 57% of patients, compared with 5 min to 72 h in 43%, and lasted longer than 72 h in 8%. Approximately 87% of patients had psychological disorders. Most patients with VM (92%) suffered from some degree of EHF hearing impairment, and 68% had SF hearing loss, which is substantially higher than their complaints (43%). Moreover, the mean EHF hearing threshold cutoff value (57 dB HL) worked well in predicting SF hearing loss (area under curve, AUC, 0.827), outperforming distortion product optoacoustic emission (AUC, 0.748).

**Conclusion:**

VM has a wide range of clinical manifestations. Hearing loss had a considerably higher rate compared to actual complaints. Moreover, patients with VM tended to have bilateral EHF and high-frequency hearing loss. The effectiveness of the mean EHF hearing threshold cutoff value in predicting hearing loss supported its use in the early detection of hearing loss and monitoring disease progression.

## Introduction

Vestibular migraine (VM) presents with a recurrence of vestibular symptoms, a history of migraine, and a temporal association between vestibular spells and migraine symptoms (Lempert et al., 2012). Its lifetime prevalence in the general population is roughly 1%, accounting for approximately 12.3% of the cases in vertigo centers (Neuhauser et al., 2006; Strupp et al., 2020). VM can develop at any age (average age 40.9 years), while it tends to afflict those with a history of migraine (Neuhauser et al., 2006) and women who are below 40 years of age, those who have anxiety or depression, and those who have previously developed sustained head trauma (Formeister et al., 2018). Patients with VM usually have central and/or peripheral vestibular and auditory dysfunction (Radtke et al., 2012).

Nevertheless, the audiological symptoms of VM are still poorly understood. Neuhauser et al. (2006) reported that 36% of patients with VM had cochlear symptoms, including tinnitus (15%), hearing loss (9%), and aural fullness (15%), during vertigo episodes. Moreover, Radtke et al. (2012) found that the presence of cochlear symptoms increased from an initial 26 to 77% after a 9-year follow-up.

Notably, hearing loss is the third leading cause of years lived with disability worldwide and affects 36.3 million people (Vos et al., 2017). Migraine is one of the leading causes of sudden sensorineural hearing loss (SSNHL), particularly in people over the age of 40 (Viirre and Baloh, 1996; Mohammadi et al., 2020). VM is associated with both peripheral and central auditory dysfunction (Xue et al., 2020). However, researchers disagreed on the features of hearing loss in VM. During attacks, 26.2 and 15.4% of patients with VM and pVM experienced hearing loss (Lopez-Escamez et al., 2014). Zhang et al. (2016) reported that only 3% of patients with VM in Chinese subjects experienced hearing loss during vertigo attacks. Radtke et al. (2012) found that patients with VM were more likely to have bilateral high-frequency hearing loss than low-frequency hearing loss. Xue et al. (2020) reported that patients with both migraine and VM were at a higher risk for low-frequency sensorineural hearing loss (SNHL). Furthermore, current treatments for VM mainly included acute pharmacologic treatment for attacks, preventive pharmacologic therapy for migraine, and vestibular rehabilitation with little attention to addressing hearing loss.

To the best of our knowledge, extended high-frequency (EHF; 8–20 kHz) hearing plays an obscure but important role in our work and daily lives, including enhancing speech perception in noise (Zadeh et al., 2019) and cognitive ability, particularly global executive function (Brännström et al., 2018). Moreover, EHF pure-tone audiometry (PTA) can be used for the early detection of hearing loss caused by ototoxic drugs (Sakamoto et al., 2000; Al-Malky et al., 2015), noise (Le Prell et al., 2013), and autoimmune diseases (Öztürk et al., 2004; González et al., 2017). Nevertheless, few studies investigated EHF (8–20 kHz) audiometry in patients with VM.

Therefore, the aim of the present study was to (1) explore the audiological features of patients with VM between standard frequencies (SFs) and EHFs, (2) determine the use of EHF audiometry as a tool for the early detection of hearing loss in patients with VM, and (3) offer a rationale for the integration of a specific audiological assessment in the differential diagnosis and treatment of VM.

## Methods

A prospective cross-sectional study was conducted in the Department of Otorhinolaryngology of a tertiary hospital. The study was approved by the Ethics Committee of Wuhan Union Hospital under code number 20210873. A detailed medical history was taken, auditory-vestibular tests and psychological assessments on the participants were performed, and these data were analyzed in detail.

### Inclusion criteria

Patients who had a definite VM (dVM) and probable VM (pVM) and attended the otolaryngology outpatient service of a tertiary medical institute between October 2021 and February 2022 were sequentially included. The diagnosis of VM was established on basis of the criteria formulated by Barany society in 2012 (Lempert et al., 2012). Existing migraine or a previous history of migraine with or without aura was assessed based on the International Classification of Headache Disorders (Olesen, 2018).

### Exclusion criteria

All patients underwent auditory, vestibular, and neurological examinations or tests, such as otoscopy, an acoustic impedance test, PTA, a glycerin test, electrocochleography, and magnetic resonance imaging (MRI). The study excluded patients who had any external or middle ear diseases, had received ear surgery, had Meniere's disease (MD), had an acoustic neuroma, had chronic exposure to noise, had hereditary deafness, had an inner ear deformity, and took ototoxic drugs (Supplementary Figure S1).

### Clinical evaluation

A predetermined set of variables was extracted from medical records, supplemented by follow-up telephone calls when necessary, and then entered into a database. Clinical variables included gender, age, vertigo attacks, hearing loss, headache or migraine features, and other accompanying symptoms (e.g., aural fullness, tinnitus, nausea, and vomiting), motion sickness, sleep disorders, allergies, autoimmune diseases, cardiovascular risk factors, anxiety, depression, and a family history of vertigo or migraine.

### Pure-tone audiometry

Pure-tone air conduction audiometry was performed separately in each ear for SFs (0.125, 0.25, 0.5, 1, 1.5, 2, 3, 4, 6, and 8 kHz) and EHFs (10, 12.5, 16, and 20 kHz) using an Astera audiometer (Otometrics A/S, Taastrup, Denmark). As ISO 389-5 2006 did not provide an equivalent reference threshold for a frequency of 20 kHz, it was excluded from the analysis. The underdetected frequency threshold was measured at 120 dB HL. The frequency could be divided into low frequency (0.125–0.5 kHz), middle frequency (1–2 kHz), high frequency (4–8 kHz), and EHF (10–16 kHz). If no response was obtained at one frequency, it was recorded as 120 dB HL. The result was taken as EHF hearing loss when the hearing threshold was greater than 20 dB HL at one or more frequencies ranging from 10 to 16 kHz in one or both ears.

A chart was prepared by comparing the results of a previous study on ETF PTA in healthy people (Wang et al., 2021). Statistical analysis (chi-squared test for independent samples) was performed to analyze the normalized percentage at 8–16 kHz. For comparison purposes, this study did not take into account the publication year or the measurement method.

### Otoacoustic emission testing

Distortion product optoacoustic emission (DPOAE) was recorded by using a cochlear emission analyzer (Otometrics A/S, Taastrup, Denmark). The frequency ratio was set to 1.22 (L1 = 70 dB SPL, L2 = 60 dB SPL). DPOAE data were recorded for different frequency regions at 0.75, 1, 2, 3, 4, 6, and 8 kHz. For all frequencies, DPOAE measurements were considered detectable when the signal-to-noise ratio was greater than 6 dB. Abnormality was defined as a decreased or undetectable reactive amplitude at one or more frequencies.

### Vestibular autorotation test

The vestibular autorotation test (VAT) was performed using a VAT device (NeuroCom International, Inc., Clackamas, OR, USA). VAT was considered abnormal when the gain, phase, or asymmetry decreased or increased.

### Psychological evaluation

All patients were psychometrically assessed to confirm the presence of anxiety, depression, and somatic symptoms.

Generalized anxiety disorder (GAD) assessment (Spitzer et al., 2006): GAD-7 consisted of seven items. Scores of 5, 10, and 15 were taken as the cutoff points for mild, moderate, and severe anxiety, respectively.

Patient health questionnaire 9 (PHQ-9) (Kroenke et al., 2001): It was used to make a preliminary diagnosis of depression. Depression severity was rated on a scale of 0–27, with points 0–4 being no depression, 5–9 being mild, 10–14 being moderate, 15–19 being moderately severe, and 20–27 being severe depression.

Somatic symptom self-rating scale (SSS) (Jiang et al., 2019): It consisted of 20 items, covering physical disorders, anxiety disorders, depression disorders, and anxiety and depression disorders. The SSS-CN scores 20–29, 30–39, 40–59, and ≥60 corresponded to normal, mild, moderate, and severe somatic symptom disorder (SSD), respectively.

### Statistical analysis

Data were entered into the EpiDate 3.1 database and then analyzed with GraphPad Prism 9. First, the distribution state of continuous variables was determined as normal or skewed. For non-normally distributed continuous variables, data distribution was described using the median and interquartile range, and difference analysis was done using the nonparametric Mann–Whitney U test; for normally distributed variables, dispersion and central tendency were represented respectively by using the mean and standard deviation (SD), and difference analysis was done using two independent sample *t*-tests. The chi-squared test was used to observe differences between dichotomous variables.

To analyze the predictive factors for hearing loss (outcome, SF hearing loss = 1, normal SF hearing = 0), the mean EHF hearing threshold and DPOAE results (abnormal = 1 and normal SF hearing = 0) were used to perform logistic regression analysis. AUC was used to evaluate the predictive power.

## Results

### Clinical manifestations

A total of 37 adult patients with VM/pVM (aged 19–68 years, with a mean age of 47.1 years, including 27 women and eight men) were enrolled in this study. Fifteen patients with pVM having a mean age of 45.13 and 22 patients with VM having a mean age of 46.68 years were recruited. The female-to-male ratio was higher in patients with VM (1.5) than in patients with pVM (6.3), but the difference between the two was not significant. There were no statistically significant differences in vertigo features between patients with VM and their pVM counterparts. For the total cohort, the median disease duration was 16 months, the median vertigo spell lasted for 2 days, and the affected median duration was 4 days per month in the past 6 months. Vertigo types in patients with both VM and pVM varied. Overall, the most common types were vestibulo-visual symptoms (83.78%) and internal vertigo (54.05%), followed by dizziness (43.24%), and the rarest was a posture symptom (10.81%). We found that, in 56.76% of patients, the vertigo attack persisted no longer than 5 min, compared with 5 min to 72 h in 43.24% of the subjects. In three patients with VM, the vertigo episode lingered for more than 72 h.

It was found that 26.67% of patients with pVM had migraine currently or earlier with or without aura, with the rate being 100% in patients with VM. Migraine features, such as, phonophobia and photophobia, were more frequent in the VM group than in the pVM group.

The cochlear symptom in the interval episode were common in patients with VM and pVM. A total of 16 participants (43.24%) complained of hearing loss in one or both ears, and 20 patients (54.05%) had unilateral or bilateral tinnitus.

The prevalence of motion sickness, phonophobia, and photophobia was higher in patients with VM than in patients with pVM. The rates of nausea and vomiting, family history, psychological disorder, autoimmune diseases, cardiovascular risk factors, and sleep disorder were frequent in both the pVM and VM groups, and no statistically significant differences in these accompanying disorders or conditions were observed (Table 1).

**Table 1:** Clinical characteristics of patients with probable vestibular migraine (pVM) and vestibular migraine (VM).

	**pVM (*n =* 15)**	**VM (*n =* 22)**	**All (*n =* 37)**
Female-to-male ratio	1.5	6.3	3.1
Mean age (SD) (years)	45.13 (13.38)	46.68 (14.31)	46.05 (13.97)
Disease duration, m (Q1, Q3), (month)	8 (1, 33)	28.5 (4.5, 72)	16 (2.75, 60)
Frequency of vertigo spell, m (Q1, Q3), (/6 months)	2 (1, 4.5d)	2.5 (1, 5.75)	2 (1, 5)
Days affected, m (Q1, Q3), (/month)	2 (0.4, 27.5)	4 (2, 10)	4 (1.8, 10)
**Vertigo type** ^a^			
Internal vertigo	60% (*n =* 9)	47.83% (*n =* 11)	54.05% (*n =* 20)
Dizziness	60% (*n =* 9)	31.82% (*n =* 7)	43.24% (*n =* 16)
Vestibulo-visual symptoms	80% (*n =* 12)	86.36% (*n =* 19)	83.78% (*n =* 31)
Posture symptoms	6.67% (*n =* 1)	13.64% (*n =* 3)	10.81% (*n =* 4)
**Duration of vertigo attacks** ^a^			
<5 min	53.33% (*n =* 8)	59.09% (*n =* 13)	56.76% (*n =* 21)
5 min−72 h	60% (*n =* 9)	31.82% (*n =* 7)	43.24% (*n =* 16)
>72 h	0 (*n =* 0)	13.64% (*n =* 3)	8.11% (*n =* 3)
Migraine diagnosis	26.67% (*n =* 4)	100% (*n =* 22)	72.97% (*n =* 27)
Phonophobia^*^	20% (*n =* 3)	54.% (*n =* 12)	40.54% (*n =* 15)
Nausea and/or vomiting	26.67 (*n =* 4)	54.55% (*n =* 12)	43.24% (*n =* 16)
Photophobia^*^	20% (*n =* 3)	54% (*n =* 12)	37.84 (*n =* 14)
Tinnitus^b^	53.33% (*n =* 8)	54.55% (*n =* 12)	54.05% (*n =* 20)
Hearing loss complaint^b^	40% (*n =* 6)	45.45% (*n =* 10)	ai% (*n =* 16)
Autoimmune diseases	13.33% (*n =* 2)	18.18% (*n =* 4)	16.22% (*n =* 6)
Cardiovascular risk factors	46.67% (*n =* 7)	50% (*n =* 11)	48.65% (*n =* 18)
Motion sickness^b*^	40% (*n =* 6)	81.82% (*n =* 18)	64.86% (*n =* 24)
Family history of vertigo/migraine	46.67% (*n =* 7)	40.91% (*n =* 9)	43.24% (*n =* 16)
Psychological disorder^b^	33.33% (*n =* 5)	36.36% (*n =* 8)	35.14% (*n =* 13)
Sleep disorder^b^	66.67% (*n =* 10)	77.27% (*n =* 17)	72.97% (*n =* 27)

^a^More than one result. ^b^In the interval episode. SD, standard deviation; m, median; Q1, quartile 1; Q3, quartile 3. ^*^p < 0.05, indicating that a statistically significant difference was found between patients with VM and pVM.

### Pure-tone audiometry

A difference in SF and EHF threshold of both ears in patients with pVM and VM was not significant (Table 2).

**Table 2:** The threshold of both ears in patients with pVM and VM at standard frequency (SF) and extended high frequency (EHF).

**Frequency (kHz)**	**pVM (*n =* 15), mean (SD) (dB HL)**	**VM (*n =* 22), mean (SD) (dB HL)**	**All (*n =* 37), mean (SD) (dB HL)**
0.125	17.83 (5.68)	16.19 (5.10)	16.77 (5.47)
0.25	16.73 (5.68)	14.24 (6.67)	15.14 (7.52)
0.5	15.96 (8.31)	14.22 (7.99)	15.06 (7.72)
1	20.00 (6.80)	16.52 (9.83)	17.97 (9.45)
2	22.12 (7.84)	19.46 (13.11)	20.65 (12.42)
4	25.38 (10.30)	21.96 (17.37)	23.41 (17.39)
8	32.31 (16.52)	26.85 (23.85)	28.91 (26.59)
10	41.04 (29.98)	35.76 (31.78)	38.21 (33.71)
12.5	58.33 (41.07)	48.70 (36.20)	52.24 (37.46)
16	108.8 (29.80)	105.87 (36.88)	107.54 (34.22)

VM, vestibular migraine; pVM, probable vestibular migraine.

Hearing loss at standard frequency (>20 dB HL; 0.125–8 kHz) was found to be common in patients with pVM/dVM. Approximately 67.57% of patients (*n* = 25) had hearing loss, which was much more than their complaint/counterpart (43.24%). In 60% of patients with pVM (*n* = 9) and 72.73% of patients with VM (*n* = 16), the threshold was unilaterally or bilaterally elevated across SFs. The rate of bilateral hearing loss at SF (68%, *n* = 17) was higher than that of unilateral hearing loss as SF (*n* = 8, 32%).

A total of 34 participants (91.89%) suffered from some degree of EHF hearing loss (>20 dB HL, 10–16 kHz), as defined by the International Organization for Standardization. Of them, 94.12% (*n* = 32) had bilateral EHF hearing loss. Patients mainly developed total hearing loss (107.54 ± 34.22) at 16 kHz and moderate to severe hearing loss at 10 and 12.5 kHz (38.21 ± 33.71 and 52.24 ± 37.46).

When analyzing thresholds at all frequencies (0.125–20 kHz) by gender, statistically significant differences were found only at 12.5 kHz but not at other frequencies (Supplementary Table S1).

**Figure 1:**
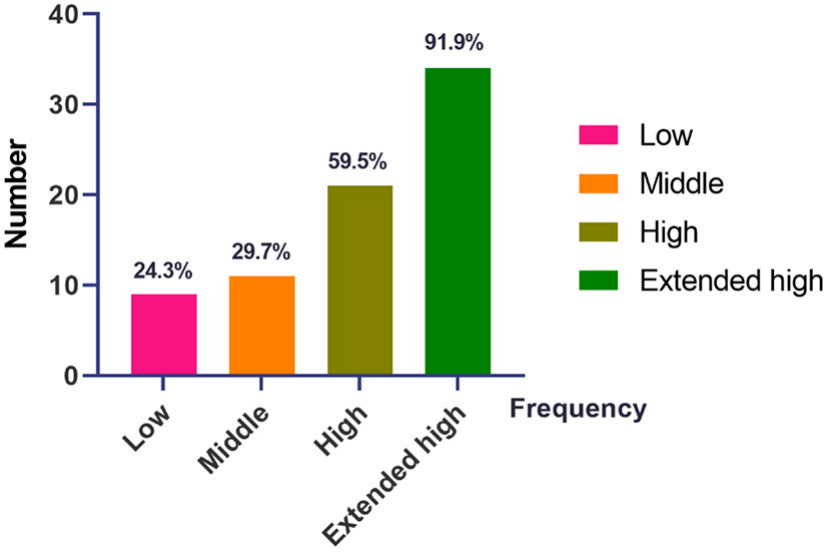
The distribution of hearing loss at different frequencies in patients with pVM/VM. VM, vestibular migraine; pVM, probable vestibular migraine. Low, low frequency, 0.125–0.5 kHz. Middle, middle frequency, 1.0–2.0 kHz. High, high frequency, 4.0–8.0 kHz. Extended high, extended high frequency (EHF), 10.0–16.0 kHz.

A large number of patients with pVM/dVM had high-frequency (59.5%) and EHF (91.9%) hearing loss as compared to low- (24.3%) and middle-frequency (24.3%) hearing loss (Figure 1).

In comparison to the study by Wang et al. (2021), no difference was found between patients who had pVM/dVM and those between the ages of 20–40, but only a significant difference was found between patients with pVM/dVM over 40 years and healthy people (Table 3 and Supplementary Tables S2, S3).

**Table 3:** A comparison of normalized percentage at 8–16 kHz from the current study with that of a previous study.

	**Frequency (kHz)**			

**Age group (years)**		**8**	**10**	**12.5**	**16**
21–30	Present study (*n =* 4)	100	100	100	75
	Wang et al. (2021) (*n =* 46)	100	100	100	100
31–40	Present study (*n =* 9)	100	100	100	33.3
	Wang et al. (2021) (*n =* 50)	100	100	100	64
41–50	Present study (*n =* 9)	100	75^**^	75	0^*^
	Wang et al. (2021) (*n =* 88)	100	100	93.2	38.6
51–60	Present study (*n =* 11)	90.9^*^	90.9^*^	72.7	0^*^
	Wang et al. (2021) (*n =* 78)	100	100	82.1	30.8
61–70	Present study (*n =* 4)	75	50^*^	25	0
	Wang et al. (2021) (*n =* 62)	100	97.4	74.2	16.1

^*^p < 0.05, ^**^p < 0.01; ^***^p < 0.001.

**Figure 2:**
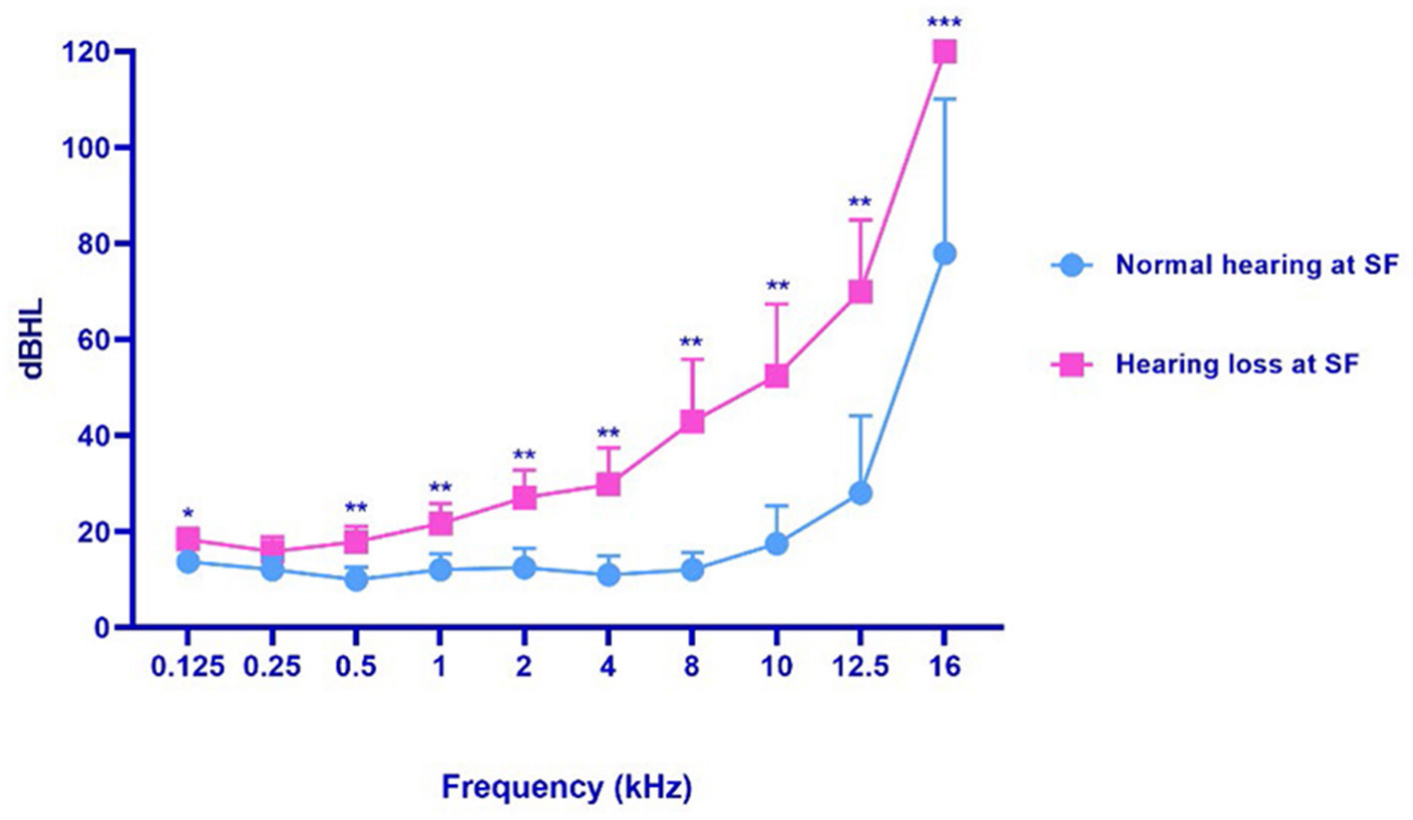
The hearing threshold of patients with SF normal hearing and patients with SF hearing loss. VM, vestibular migraine, including definite and probable vestibular migraine. SF, standard frequency (0.125–8 kHz). *p < 0.05, **p < 0.01; ***p < 0.001.

In terms of SF hearing loss, subjects in our cohort could be further divided into two subgroups, patients with VM who had normal SF hearing and those who had SF hearing loss. In addition, we then selected the ear with the more severe hearing loss to represent the patient's hearing condition. We found that the subgroup with SF hearing loss had poor SF hearing as well as EHF hearing (Figure 2). Moreover, there was a more remarkable difference with the frequency increasing from low to extended high levels (Table 4).

**Table 4:** The frequency characteristics of hearing threshold in patients with VM with SF normal hearing and patients with SF hearing loss.

**Frequency**	**Group with SF hearing loss**	**Group with SF normal hearing**	***p***
Low, mean (SD)	17.38 (6.55)	11.94 (3.11)	0.012096
Middle, mean (SD)	24.38 (11.51)	12.29 (5.35)	0.001945
High, mean (SD)	36.35 (23.72)	11.67 (4.12)	0.001403
Extended high, mean (SD)	80.83 (22.92)	41.25 (27.24)	0.000126

VM, vestibular migraine; including definite and PVM. SF, standard frequency (0.125–8 kHz). Low frequency, 0.125–0.5 kHz. Middle frequency, 1.0–2.0 kHz. High frequency, 4.0–8.0 kHz. EHF, 10.0–16.0 kHz. SD, standard deviation.

In terms of the presence or absence of tinnitus, patients could be classified into VM with tinnitus and VM without tinnitus. The group of VM with tinnitus consisted of 20 patients (54.1%), and the group of VM without tinnitus consisted of 17 patients (45.9%). VM with tinnitus had a slightly higher SF and EHF threshold than VM without tinnitus, but the difference between the two was not significant (Supplementary Table S4). Patients with VM who had tinnitus and those who had no tinnitus had hearing loss principally at 2–16 kHz.

### Distortion product optoacoustic emission

Seven patients with pVM and 15 patients with VM both had abnormal DPOAE in one or both ears (Supplementary Table 5S). In contrast to the result of SF and EHF PTA, the DPOAE abnormal rate from low frequency to EHF remained the same, while during impairment it progressed severely with the frequency increasing from the lower to higher level.

### Vestibular autorotation test

Twenty-seven patients had VM and seven patients had an abnormal VAT. In patients with abnormal VAT (*n* = 27), approximately 66.7% (*n* = 18) had central vestibular disorder, 11.1% (*n* = 3) had peripheral vestibular dysfunction, and 10.8% (*n* = 4) had vestibular impairment of unknown origin.

### Psychological assessment

Approximately 86.5% (*n* = 32) of patients were found to have much more psychological disorder than their complaint (35.14%). In our cohort, 37.8% (*n* = 14) were suffering from depression, 32.4% (*n* = 12) from anxiety, and 70.3% (*n* = 26) from somatic symptoms. The psychological disorders were mostly mild to moderate in nature (Supplementary Table S6).

### Magnetic resonance imaging

Magnetic resonance imaging revealed no characteristic findings in patients with VM. Some non-specific findings were inclusive of a close correlation between the facial auditory nerve and peripheral small blood vessels and lacunar cerebral infarction.

### The mean EHF threshold, the more powerful predictive diagnostic factor for SF hearing loss

For those patients for whom the EHF threshold was evaluated, 81.48% of them had an elevated threshold at SF. In patients with pVM/dVM and unilateral SF hearing loss, there was a constant increase in EHF threshold.

Both mean EHF and DPOAE hearing thresholds were risk factors for SF hearing loss. Their effect on the probability of hearing loss occurring is shown in the following formulas: *P* = −4.182 + 0.07325*X*_*EHF*_, *P* = −1.946 + 3.290*X*_*DPOAE*_.

**Figure 3:**
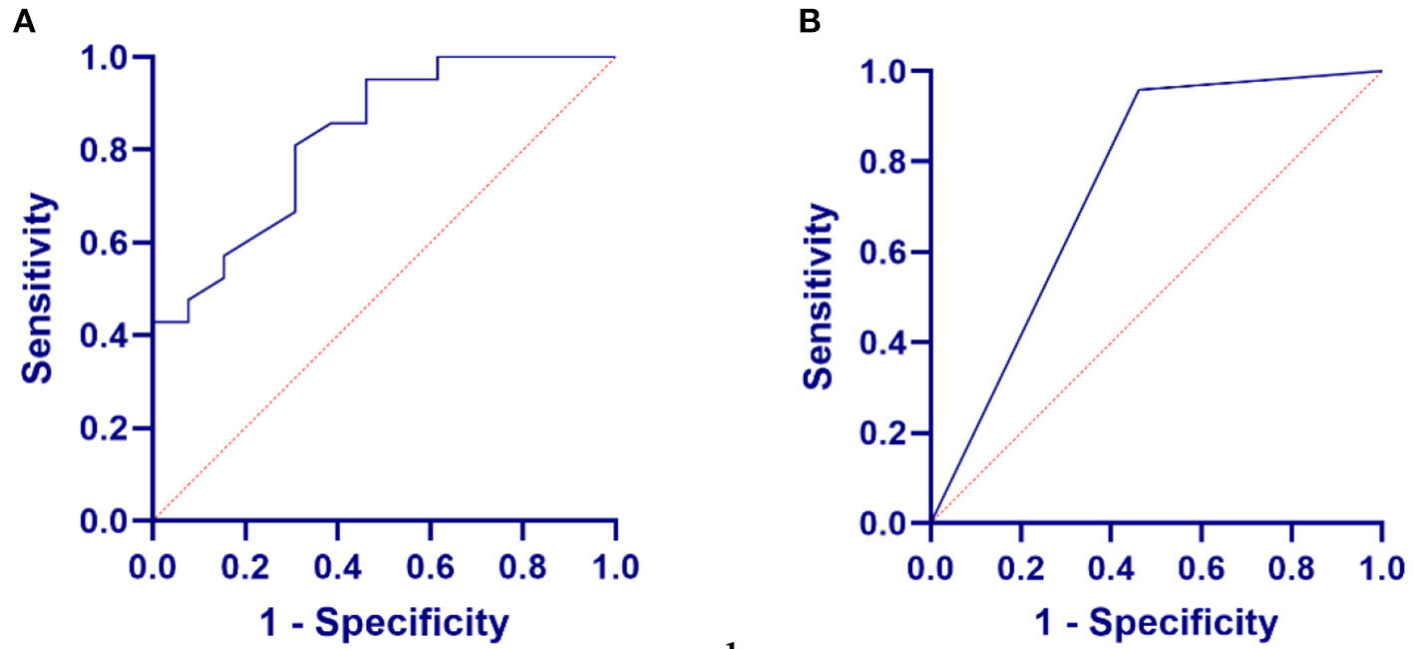
Relationship between EHF pure-tone test or distortion product optoacoustic emission (DPOAE) in VM and SF hearing loss. **(A)** The relationship between the mean EHF hearing threshold in VM and SF hearing loss. **(B)** The relationship between the DPOAE in VM and SF hearing loss.

Then, the receiver operating characteristic (ROC) curve analysis revealed that another cutoff value of >57.09 dB HL of the mean EHF hearing threshold had maximal sensitivity and specificity for predicting poor hearing [ROC curve area, 0.827 (95% confidence interval (CI), 0.688–0.968); *p* = 0.0015] (Figure 3A). Moreover, the predictive power for SF hearing loss of the mean EHF hearing threshold was greater than that for DPOAE [ROC curve area, 0.748, (95%CI, 0.5638–0.9330); *p* = 0.0137] (Figure 3B).

### Clinical features according to the mean EHF hearing threshold

In terms of EHF mean hearing threshold, our patients were classified into two groups: patients with low mean EHF hearing threshold (LEHF) and those with high mean EHF hearing threshold (HEHF). Statistically significant differences were found in several clinical features between the two groups (Table 5). Patients with HEHF were older, and most of them had more severe SF hearing loss than their LEHF counterparts. Moreover, the HEHF group had a greater number of patients with pVM.

**Table 5:** Clinical characteristics of patients with low mean EHF hearing threshold (LEHF) and those with high mean EHF hearing threshold (HEHF).

	**Patients with LEHF (*n =* 10)**	**Patients with HEHF (*n =* 27)**
Female-to-male ratio	9	2.375
Mean age (SD) (years)^*^	34.2 (10.51)	50.44 (12.45)
Disease duration, m (Q1, Q3), (month)	31.25 (0.53, 84)	16 (4, 48)
Frequency of vertigo spell, m (Q1, Q3), (/6 months)	1.5 (1, 5.25)	2 (1, 5.25)
Days affected, m (Q1, Q3), (/month)	6.5 (2, 13.75)	3 (1, 10)
**Vertigo type** ^a^		
Internal vertigo	50% (*n =* 5)	59.09% (*n =* 15)
Dizziness	30% (*n =* 3)	48.15% (*n =* 13)
Vestibulo-visual symptoms	70% (*n =* 7)	88.89% (*n =* 24)
Posture symptoms	10% (*n =* 1)	11.11% (*n =* 3)
**Duration of vertigo attacks** ^a^		
<5 min	70% (*n =* 7)	59.09% (*n =* 15)
5 min−72 h	30% (*n =* 3)	48.15% (*n =* 13)
>72 h	0 (*n =* 0)	11.11% (*n =* 3)
VM^*^	90% (*n =* 9)	48.15% (*n =* 13)
pVM^*^	10% (*n =* 1)	51.85% (*n =* 14)
Phonophobia	40% (*n =* 5)	37.04% (*n =* 10)
Nausea and/or vomiting	30% (*n =* 3)	48.15% (*n =* 13)
Photophobia	40% (*n =* 4)	37.03% (*n =* 10)
Tinnitus^b^	60% (*n =* 6)	51.85% (*n =* 14)
Hearing loss complaint^b^	20% (*n =* 2)	51.85% (*n =* 14)
Autoimmune disease	10% (*n =* 1)	18.52% (*n =* 5)
Cardiovascular risk factors	30% (*n =* 3)	55.56% (*n =* 15)
Motion sickness^b^	90% (*n =* 9)	55.56% (*n =* 15)
Family history of vertigo/migraine	30% (*n =* 3)	48.15% (*n =* 13)
Psychological disorder^b^	60% (*n =* 6)	81.48% (*n =* 22)
Sleep disorder^b^	70% (*n =* 7)	74.07% (*n =* 20)
Abnormal PTA at SF^*^	30% (*n =* 3)	81.48% (*n =* 22)
Abnormal VAT	60% (*n =* 6)	77.78% (*n =* 21)
Abnormal DPOAE	50% (*n =* 5)	55.56% (*n =* 17)

^a^More than one result. ^b^In the interval episode. ^*^p < 0.05, indicating a statistically significant difference between patients with VM and pVM.

## Discussion

First, this study showed that VM had a wide variety of clinical manifestations, and some of its clinical features were inconsistent with previously reported characteristics. Secondly, we found that auditory system dysfunction, especially hearing loss, was common but less explicable in pVM/dVM. In patients with dVM/pVM, hearing loss was characterized by bilateral involvement and greater severity, particularly at EHF and high frequency, followed by middle and low frequencies. Moreover, we identified the mean EHF threshold as a better predictor for poor SF hearing. Patients with VM could be classified into two groups, namely the LEHF and HEHF groups, in terms of the cutoff value of 57 dB HL of the mean EHF threshold.

Vestibular migraine had clinical manifestations, which varied substantially. Women and people at the age of 40 were preponderant, which coincided with previous findings (Formeister et al., 2018). VM had a robust trend toward familial clustering (Requena and Espinosa-sanchez, 2014). A family history of migraine and/or vertigo was common in patients with VM (43.24%) but less common than previously reported (Teggi et al., 2018). Teggi et al. (2018) found that approximately 70.2% of patients with VM had a positive family history of migraine and 66.3% had a family history of vertigo. This difference might be attributed to the relatively small sample size of our cohort and racial differences.

Similar to other studies, our study also found that the features of migraine and vertigo showed great variations in terms of type, duration, attack frequency, and the mean number of days affected per month (Radtke et al., 2012; Dieterich et al., 2016; Teggi et al., 2018). The type and duration of vertigo differed considerably across numerous studies. Patients with dVM and pVM presented similarly in our cohort. As a result, dizziness and vestibulo-visual symptoms (83.78%) and internal vertigo (54.05%) were the major manifestations, followed by dizziness (43.24%) and posture symptoms (10%). Meanwhile, Teggi et al. (2018) reported that internal vertigo (73%) and postural symptoms (61.5%) were the most common symptoms in dVM, followed by spontaneous dizziness (47.2%) and external vertigo (25%). However, according to one study, unsteadiness (91%), balance problems (82%), and vertigo (57%) were the most common symptoms (Cohen et al., 2011). To date, what are the major and characteristic presentations of vertigo in VM remain a head-scratching issue among otorhinolaryngologists. Consistent with previously published data, our study found that the duration of vertigo attacks was highly variable, but the vertigo episode lasted for less than 5 min in 56.76% of patients with VM, as opposed to the much lower rate of 21% reported by other researchers (Teggi et al., 2018). According to the diagnostic criteria for the VM state, 30% of patients with VM experienced attacks that lasted only minutes and 10% experienced attacks that lasted only seconds (Lempert et al., 2012), with the rates being much lower than our findings. We speculated that patients with VM who visited the department of otolaryngology might present vestibular symptoms different from those who visited the emergency room or neurology clinics, and, reportedly, many patients with VM might have experienced vertigo spells that lasted less than 5 min. Therefore, to accurately identify patients with VM, special attention should be paid to the duration of a vertigo attack in patients with VM.

Regarding the comorbidities of VM, sleep disorders, anxiety, depression, and somatic conditions, cardiovascular risk factors, and autoimmune diseases were reportedly common, on par with migraine (Macgregor et al., 2011). Some researchers even proposed that migraine-anxiety-related dizziness was a symptom complex of balance disorder, migraine, and anxiety (Furman, 2005). Comorbidities of VM warrant further research and are important to VM therapy.

Migraine raises serious concerns because it is an important risk factor for sudden hearing loss (Viirre and Baloh, 1996; Dash et al., 2008; Hwang et al., 2018), and auditory system dysfunction was found to be more common in pVM/dVM than what was was previously thought. This study found accompanying cochlear symptoms in the interval episode, which was in line with prior studies (Radtke et al., 2012; Lopez-Escamez et al., 2014; Teggi et al., 2018; Benjamin et al., 2022). Notably, 74.3% of patients with VM had abnormal SF PTA, which was higher than the rates reported in other studies (Dash et al., 2008; Radtke et al., 2012). The rate of bilateral involvement was higher in our cohort than that in other studies. This phenomenon indicated that a large number of patients with VM suffered from hearing loss, which is contrary to what was previously considered. Similar to earlier findings, our subjects reported tinnitus and hearing loss rates of 42.8 and 45.7%, respectively (Radtke et al., 2012).

Hearing loss in patients with VM is frequency-specific but remains controversial. In some studies, patients with VM developed mild and reversible low-frequency hearing loss (Hwang et al., 2018; Xue et al., 2020). Xue et al. (2020) found that low-frequency hearing was more likely to be involved in VM and proposed that a history of migraine might be the cause of sudden low-frequency hearing loss. Nonetheless, our study found that SF, middle-frequency, and high-frequency (2–8 kHz) hearing loss were the most common and aggravated, and low-frequency hearing loss was much less common and much milder. Radtke et al. (2012) and Lai et al. (2019) also reported that patients with VM developed high-frequency hearing loss. Our findings can help in differentiating VM from other vestibular disorders, such as MD. Patients with MD have low-frequency hearing loss, and hearing loss can progress from low- to middle-to-high frequency hearing loss, ultimately deteriorating into pantonal hearing loss (Lopez-Escamez et al., 2015). However, patients with VM tend to have bilateral EHF and high-frequency hearing loss, followed by middle- and low-frequency hearing loss. Our findings support the notion that hearing loss in VM tends to be at high frequency, which contributes to disease differentiation.

Moreover, this was the first study to comprehensively test the EHF hearing threshold in patients with VM. We found that 91.89% of patients had an elevated threshold at one or more frequencies, especially at 16 kHz and subsequently at 12.5 kHz. An examination of the function of outer hair cells revealed that EHF had a higher rate of abnormality than PTA (74.3%) and DPOAE (62.9%),. This discrepancy could be explained by central and peripheral auditory system damage in VM: (1) reversible vasospasm of the internal auditory artery or its branches (Viirre and Baloh, 1996; Baloh, 1997; Lai et al., 2019); (2) some inflammation and vasoactive neuropeptides (e.g., substance P, 5-HT, and GCRP) affect the activity of sensory fibers innervating the inner ear and central auditory system (Cabanillas and Luebke, 2002; Vass et al., 2004; Koo and Balaban, 2006); (3) VM responds to abnormal brain sensitization, which results in a disordered multimodal sensory integration and processing involving the pain matrix, vestibular pathways, auditory pathways, etc. (Shin et al., 2014; Espinosa-Sanchez and Lopez-Escamez, 2015); (4) several genetic variants of ionic channels and receptors have been identified to be associated with migraine, both in the brain and inner ear, such as CACNA1A (Wiest et al., 2001), and neuronal voltage-gated calcium channels (Cav2.1) (Moskowitz et al., 2004). Furthermore, the different cochlea parts were vulnerable in different ways. Wu et al. (2018) reported that the gene Calca/Cgrpα was highly expressed in type II afferent neurons as compared to type I ones after hearing the onset. Furthermore, Calca/Cgrpα drives reporters preferentially in “higher frequency” type II SGNs near the cochlear base. CGRP is a neurotransmitter that plays an important role in the development of migraine. Ying et al. found an innate apical-to-basal gradient of decreasing SOD2 expression in mammals in the absence of an ototoxic challenge. It might suggest a selection bias in the evolutionary process of cochlear design that favored higher SOD2 expression in the apex, corresponding to higher ROS load in the apical cochlear turn but lower response capacity at the cochlear base, contributing to cumulative susceptibility to high-frequency hearing loss. Therefore, cochlear basal turns, such as high-frequency and EHF hearing, were more vulnerable than low-frequency hearing. Ying and Balaban (2009) found an innate apical-to-basal gradient of decreasing SOD2 expression in mammals in the absence of ototoxic challenge. It might suggest a selection bias in the evolutionary process of a cochlear design that favored higher SOD2 expression in the apex, corresponding to higher ROS load in the apical cochlear turn, but lower response capacity at the cochlear base, contributing to cumulative susceptibility to high-frequency hearing loss. Therefore, cochlear basal turns, such as high-frequency and EHF hearing, had more vulnerability than low-frequency hearing. The combination of vasodilatory and contractile activity, neuroinflammation, and neuroexcitatory plasticity during the recurrence of a VM attack led to such a vulnerability.

We further explored the risk factors for hearing loss in VM. Our results indicated that the mean EMF hearing threshold could be used to predict SF hearing loss. Moreover, the cutoff value of 57 dB HL (the mean EHF hearing threshold) outperformed DPOAE in predicting poor SF hearing. Therefore, EHF PTA might detect potential hearing damage in VM to allow for early intervention. Our study also showed that EHF PTA could be used to monitor disease progression and evaluate treatment efficacy. Hearing function was related to life quality, drop risk, and cognitive ability (Wang et al., 2022); therefore, those with an elevated EHF hearing threshold, especially those with HEHF, should receive multimodal migraine prophylaxis therapy and hearing protection to evaluate the quality of life, such as prophylactic migraine drugs, and noise avoidance.

To date, few studies focused on the differences in clinical features between patients with VM and hearing loss and those without. In this study, we found that patients with HEHF were older, but the disease duration was not different between the two groups. The EHF hearing threshold would be physically evaluated with age. The presence of VM might accelerate the progression of age-related hearing loss. In addition, when compared to patients with dVM, those with pVM were more likely to have HEHF. This might be attributed to the fact that the pVM organs involved were less vestibule-specific.

There are several limitations to this study. Achieving homogeneity was a challenge, as both dVM and pVM were included. Above all, the evaluation of familial cases of vertigo (and their diagnoses) and the clinical history of migraine precursors might present some uncertainties, as these data were only collected during a structured interview of the patient. Finally, our sample size was not sufficient, and there was no control group to allow for a more in-depth analysis and the identification of additional risk factors for hearing loss.

## Conclusion

Vestibular migraine has a wide range of clinical manifestations, including core symptoms (vestibular symptoms and migraine features), accompanying symptoms (auditory symptoms), and comorbidities (cardiovascular risk factors, sleep disorders, and psychological disorders). Auditory symptoms, especially hearing loss, were more common than previously thought. The majority of the patients with VM (74.3%) had SF hearing loss (0.125–8.0 kHz) and 97.1% had EHF hearing loss (8.0–20 kHz). Patients with VM tend to have bilateral EHF and high-frequency hearing loss (4.0–8 kHz). Moreover, EHF PTA may be used to predict hearing loss, monitor disease progression, and evaluate treatment efficacy. Patients with a high mean EHF hearing threshold (>57 dB HL) may benefit from positive multimodal migraine prophylaxis therapy and should put on hearing protection.

## Data availability statement

The raw data supporting the conclusions of this article will be made available by the authors, without undue reservation.

## Ethics statement

The studies involving human participants were reviewed and approved by the Ethics Committee of Wuhan Union Hospital. The patients/participants provided their written informed consent to participate in this study.

## Author contributions

SZ, ZG, and WK designed the research and directed its implication. ZG drafted and modified the manuscript. ZG, ET, JW, and JC contributed to the medical record collection. All authors have read and agreed to the published version of the manuscript.
