# Supplementary material for: Early detection and monitoring of hearing loss in vestibular migraine: Extended high-frequency hearing
Source: Front Aging Neurosci. 2023 Jan 10;14:1090322. doi: 10.3389/fnagi.2022.1090322 (PMC9871761; doi:10.3389/fnagi.2022.1090322)
Supplement: Supplementary file 1 [file Data_Sheet_1.docx]

Supplement figure 1 A flowchart of subjects in the present study


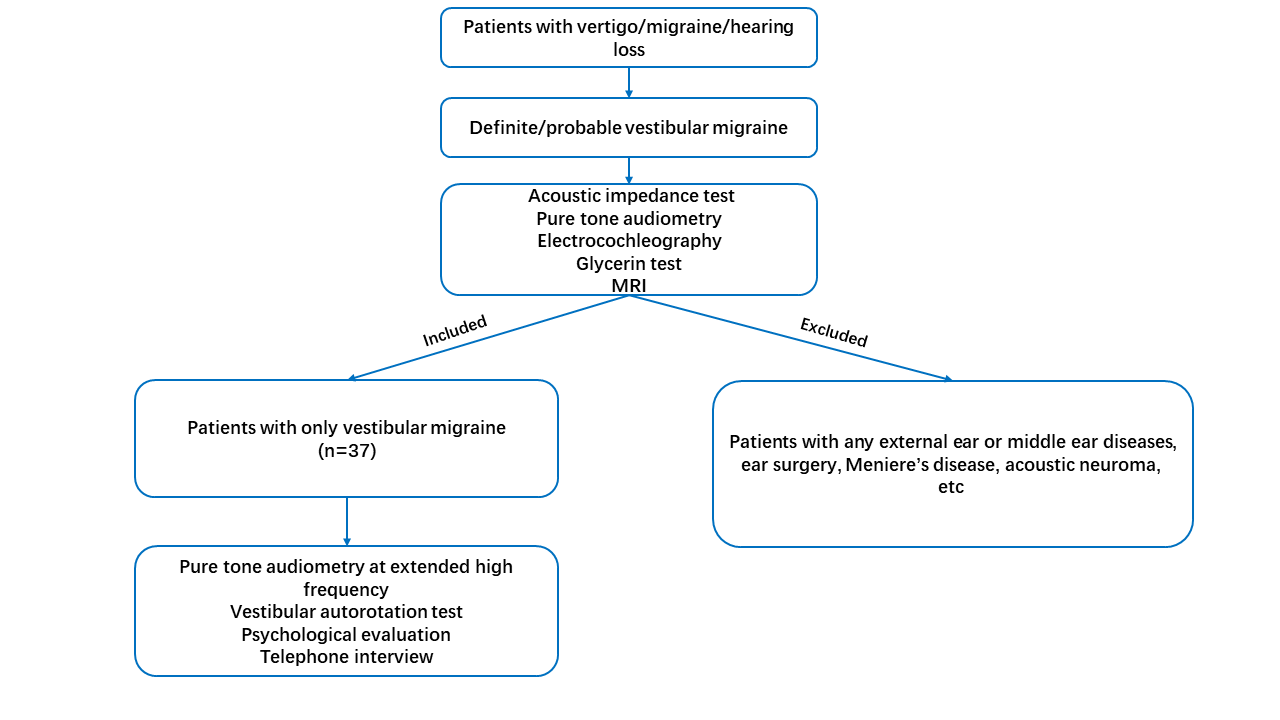


Patients were eligible for enrollment if they had received a clinical diagnosis of definite or probable vestibular migraine according to the 2012 Bárány Society and the International Headache Society criteria. Patients were excluded from the study if they had any external ear or middle ear diseases, had received ear surgery, were at other risks of developing sensorineural hearing loss or vertigo, such as Meniere’s disease, acoustic neuroma, chronic exposure to noise, hereditary deafness, inner ear deformity and had taken ototoxic drugs. Between October 2021 and February 2022, we recruited 37 patients with vestibular migraine and perform telephone interview, pure tone audiometry at extended high frequency, vestibular autorotation test, psychological evaluation.

Supplement table 1 Hearing thresholds in male group and female group.

| Frequency (kHz) | Male (n=9), mean (SD)  medians (Q1;Q2) | Female (n=28), mean (SD)  medians (Q1;Q2) | P |
| --- | --- | --- | --- |
| 0.125 | 13.89 (3.93)  15 (10;15) | 17.71 (5.40)  15 (15;20) | 0.07 |
| 0.25 | 12.78 (2.48)  15 (10;15) | 15.19 (7.51)  15 (10;17.5) | 0.37 |
| 0.5 | 15.56 (7.24)  10 (10;20) | 15.15 (7.85)  15 (10, 19.5) | 0.93 |
| 1 | 20 (11.79)  15 (10;25) | 17.96 (9.05)  20 (10;20) | 0.68 |
| 2 | 23.33 (16.16)  15 (15;35) | 21.85 (12.78)  20 (15;25) | 0.72 |
| 4 | 30 (20.41)  25 (15;50) | 21.48 (16.26)  15 (10;25) | 0.33 |
| 8 | 41.67 (25.39)  40 (15;65) | 29.63 (30.40)  20 (10;32.5) | 0.24 |
| 10 | 51.88 (32.11)  47.5 (32.5;57.5) | 36.54 (34.07)  27.5 (15;45) | 0.12 |
| 12.5 | 83.75 (39.03)  92.5 (60;120) | 46.54 (33.56)  45 (21.25;60) | 0.02* |
| 16 | 108.75 (29.76)  120 (120;120) | 104.04 (37.85)  120 (120;120) | 0.73 |

* Significant difference (p < 0.05).

Supplement table 3 Comparison of hearing thresholds at 8-16kHz between the current study and a previous study.

| Hearing threshold [dB HL] in medians (Q1;Q2) | | | | | |
| --- | --- | --- | --- | --- | --- |
| Age groups | | 8kHz | 10kHz | 12.5kHz | 16kHz |
| 21-30y | Present study (n=4) | 10.00 (5.00;16.25) | 12.50 (8.75;16.25) | 10.00 (3.75;22.5) | 5.00 (2.5;17.5) |
|  | Wang M, 2021 (n=46) | 10.00 (6.50;10.00) | 10.00 (10.00;15.00) | 15.00 (10.00,20.00) | 25.00 (10.00;45.00) |
| 31-40y | Present study (n=9) | 20.00 (10.00;25.00) | 27.50 (16.25;36.25) | 42.50 (15;48.75) | 30.00 (27.5;32.5) |
|  | Wang M, 2021 (n=50) | 15.00 (5.00,30.00) | 30.00 (12.50;45.00) | 50.00 (25.00,65.00) | 50.00 (35.00;55.00) |
| 41-50y | Present study (n=8) | 32.50 (13.75;46.25) | 25.00 (16.50;30.00) | 32.50 (21.5;43.5) | NA |
|  | Wang M, 2021 (n=88) | 20.00 (10.00,25.00) | 32.50 (20.00;50.00) | 55.00 (35.00,75.00) | 55.00 (50.00;60.00) |
| 51-60y | Present study (n=11) | 17.50 (10.00;35.00) | 40.00 (17.50;45.00) | 60.00 (51.25;65) | NA |
|  | Wang M, 2021 (n=78) | 40.00 (15.00,60.00) | 60.00 (28.75;75.00) | 65.00 (51.25;83.75) | 55.00 (55.00,60.00) |
| 61-70y | Present study (n=4) | 65.00 (55.00;75.00) | 70.00 (65.00;75.00) | NA | NA |
|  | Wang M, 2021 (n=62) | 50.00 (35.00;60.00) | 70.00 (60.00;80.00) | 55.00 (55.00,60.00) | NA |

Q1 25th empirical quartile; Q2 75th empirical quartile

NA, not applicable since the response was equivalent or smaller than 20%.

Supplement table 4 Auditory thresholds of vestibular migraine patients with and without tinnitus

| Frequency (kHz) | VM with tinnitus, mean (SD) (n=20) | VM without tinnitus, mean (SD) (n=17) | P |
| --- | --- | --- | --- |
| 0.125 | 16.94 (6.04) ) | 16.33 (4.27) | 0.75 |
| 0.25 | 15.25 (7.82) | 14.41 (4.5) | 0.62 |
| 0.5 | 16.00 (9.66) | 16.00 (6.63) | 0.56 |
| 1 | 18.68 (12.12) | 18.24 (6.4) | 0.90 |
| 2 | 22.00 (14.87) | 22.65 (11.77) | 0.89 |
| 4 | 22.37 (19.01) | 25.00 (16.18) | 0.67 |
| 8 | 35.53 (30.56) | 29.41 (28.33) | 0.55 |
| 10 | 40.83 (33.13) | 39.38 (35.44) | 0.91 |
| 12.5 | 56.39 (39.61) | 54.10 (36.79) | 0.86 |
| 16 | 104.00 (33.66) | 107.50 (33.49) | 0.82 |

Supplement table 5 Distortion product optoacoustic emission of patients with VM.

| Frequency (kHz) | Total abnormal rate | Low amplitude | Undetected rate |
| --- | --- | --- | --- |
| 0.75 | 75% | 52.5% | 22.5% |
| 1.0 | 70% | 42.5% | 27.5% |
| 1.5 | 62.5% | 37.5 | 25% |
| 2.0 | 52.5% | 20% | 32.5% |
| 3.0 | 67.5% | 30% | 37.5% |
| 4.0 | 77.5% | 40% | 37.5% |
| 6.0 | 67.5% | 30% | 37.5% |
| 8.0 | 55% | 5% | 50% |

VM, vestibular migraine, including definite and probable vestibular migraine.

Supplement table 6 Psychological assessment of patients with dVM and pVM

| Self-rating scale | pVM (n=15) | VM (n=22) | Total (n=37) |
| --- | --- | --- | --- |
| PHQ-9 (mean±SD) | 5.23±5.21 | 5.29±4.77 | 5.27±4.92 |
| GAD-7 (mean±SD) | 3.69±2.97 | 4.38±3.64 | 4.14±3.43 |
| SSS (mean±SD) | 34.62±10.89 | 35.46±11.53 | 35.16±11.31 |

dVM, definite vestibular migraine. pVM, probable vestibular migraine.

PHQ9, patient health questionnaire 9.

GAD7, generalized anxiety disorder assessment 7.

SSS, somatic symptom self-rating scale.
